# Muscle Metastasis from Undifferentiated (Anaplastic) Thyroid Carcinoma

**DOI:** 10.5334/jbsr.1604

**Published:** 2019-05-08

**Authors:** Christophe Valkenborgh, Laurent Médart, Laurent Collignon

**Affiliations:** 1 Department of Radiology, CHR Liège, BE

**Keywords:** muscle metastasis, anaplastic thyroid carcinoma

## Case Report

**Figure 1:**
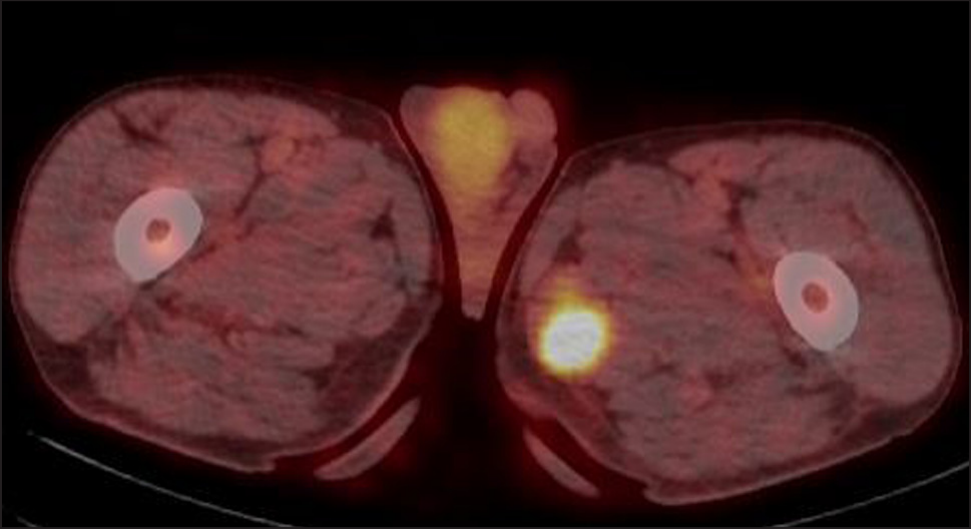


**Figure 2:**
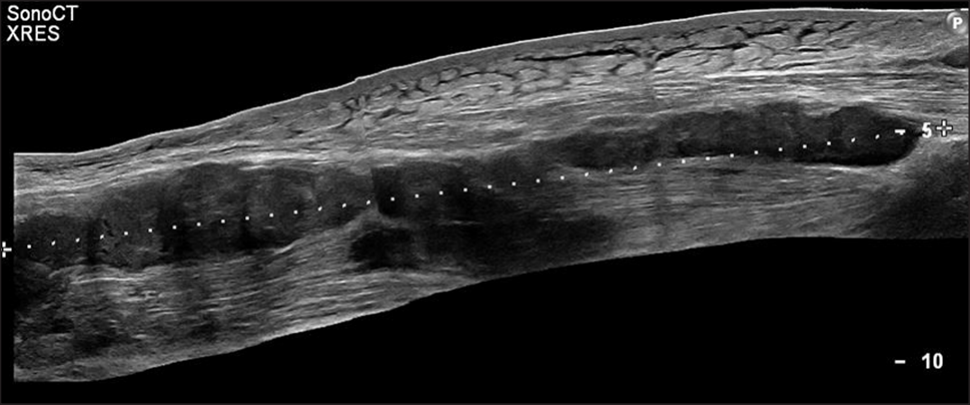


A 69-year-old man presented to the emergency department, referred by his general practitioner. He complained of tumefaction and right hemi-cervical pain, dysphagia for solids, dysphonia, and loss of weight (10 kg in two months). Blood analysis revealed an inflammatory syndrome without hyperleukocytemia. Neck computed tomography (CT) showed a mass arising from the right lobe of the thyroid focally invading the trachea, associated with esophageal extrinsic compression and bilateral cervical lympadenopathies. Open surgical biopsy led to the diagnosis of unresectable anaplastic thyroid carcinoma. Subsequent positron emission tomography (PET)-CT was performed to evaluate the extension of the neck tumor and revealed a 18-Fluoro-deoxy-glucose (FDG)-avid lesion in the left adductor space (Figure 1). Ultrasound-guided biopsy of the hypo-echoic thigh muscular mass (Figure 2) confirmed metastasis.

**Figure 3:**
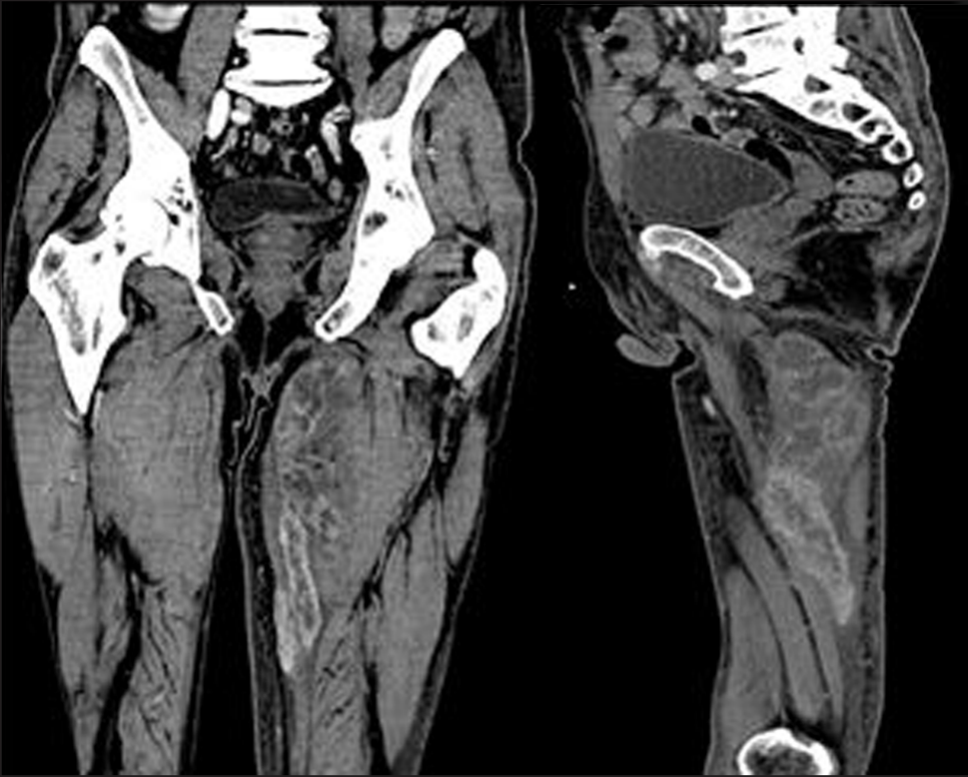


A treatment by combined radio-chemotherapy was initiated. A temporary tracheal prosthesis was positioned; dysphagia was finally handled by gastrostomy. Cervical evolution was excellent under treatment, but muscle metastasis progressed (Figure 3) and pulmonary metastasis appeared, leading to second-line chemotherapy.

## Discussion

Anaplastic thyroid carcinoma and muscle metastasis (MM) are rare. The prevalence of MM is around 1.5% of cancer in radiological series; this low prevalence is the result of several muscular protective mechanisms against metastatic invasion: contractile activity, pH variation, intramuscular blood pressure, local temperature, and production of biochemical anti-tumour factors. MM recognition and frequency are nevertheless increasing, as PET-CT detects more easily secondary muscular lesions and the overall incidence of cancer cases increases with the ageing population and the prolonged survival of cancer victims.

MM 1 are from lung cancer (25.1%), gastrointestinal tumors (21.0%), or urological tumors (13.2%). Only 3.7% of MM are from thyroid gland primary. The thigh muscles are the most frequent localization of MM (22.1%), followed by the extraocular musculature (15%) and the gluteal and paravertebral muscles (respectively 10.7% and 10.3%). Some localizations may be suggestive of a particular type of primary malignancy (e.g. breast’s metastasis in extraocular muscles).

MM are almost always hypoechoic on ultrasound, whereas there are five different patterns on CT, often related to the characteristics of the primary tumor:

– type 1 (46.5%): round or oval mass with homogeneous contrast enhancement.
– type 2 (27.7%): abscess-like with central low attenuation and rim enhancement.
– type 3 (18.1%): diffuse infiltration with muscle swelling and inhomogeneous enhancement (Figure 3).
– type 4 (6.5%): multiple intramuscular calcifications.
– type 5 (1.2%): intramuscular bleeding.

On magnetic resonance imaging, MM are typically hyperintense on T2-weighted images and of homogenous but variable signal intensity on T1-weighted images compared to the unaffected. On PET-CT, MM present as focal abnormal intramuscular uptake.
